# Unbiased phenotypic identification of functionally distinct hematopoietic progenitors

**DOI:** 10.1186/s40709-019-0097-7

**Published:** 2019-07-18

**Authors:** Grigorios Georgolopoulos, Mineo Iwata, Nikoletta Psatha, Minas Yiangou, Jeff Vierstra

**Affiliations:** 1 Altius Institute for Biomedical Sciences, Seattle, WA 98121 USA; 2 Department of Genetics, Development and Molecular Biology, School of Biology, Aristotle University of Thessaloniki, 541 24 Thessaloniki, Greece

**Keywords:** Hematopoietic progenitors, Immunophenotypic identification, Functional characterization, Single-cell, Index sorting

## Abstract

**Background:**

Hematopoiesis is a model-system for studying cellular development and differentiation. Phenotypic and functional characterization of hematopoietic progenitors has significantly aided our understanding of the mechanisms that govern fate choice, lineage specification and maturity. Methods for progenitor isolation have historically relied on complex flow-cytometric strategies based on nested, arbitrary gates within defined panels of immunophenotypic markers. The resulted populations are then functionally assessed, although functional homogeneity or absolute linkage between function and phenotype is not always achieved, thus distorting our view on progenitor biology.

**Method:**

In this study, we present a protocol for unbiased phenotypic identification and functional characterization which combines index sorting and clonogenic assessment of individual progenitor cells. Single-cells are plated into custom media allowing multiple hematopoietic fates to emerge and are allowed to give rise to unilineage colonies or mixed. After colony identification, lineage potential is assigned to each progenitor and finally the indexed phenotype of the initial cell is recalled and a phenotype is assigned to each functional output.

**Conclusions:**

Our approach overcomes the limitations of the current protocols expanding beyond the established cell-surface marker panels and abolishing the need for nested gating. Using this method we were able to resolve the relationships of myeloid progenitors according to the revised model of hematopoiesis, as well as identify a novel marker for erythroid progenitors. Finally, this protocol can be applied to the characterization of any progenitor cell with measurable function.

**Electronic supplementary material:**

The online version of this article (10.1186/s40709-019-0097-7) contains supplementary material, which is available to authorized users.

## Background

Hematopoiesis has long served as a paradigm for modelling cell development [1]. Delineation of the progenitor hierarchy and their relationships provide essential knowledge towards our understanding of cell differentiation and lineage commitment. Isolation and characterization of hematopoietic stem and progenitor cells (HSPCs) has historically relied on flow-cytometric fractionation using complex hierarchical gates of pre-defined cell-surface marker panels. The identified populations are then subjected to transplantation or in vitro clonogenic assays and a functional identity is assigned a posteriori [2–4]. Although these methods have provided tremendous insight into the biology of hematopoietic progenitors, their between relationships as well as their phenotypic definition and function are still debated and constantly revised upon introduction of updated surface marker panels [5–9]. It is now well established that under certain circumstances phenotype is decoupled from function as culture conditions or donor age can disrupt this relationship by preserving phenotype but altering or impairing function [10, 11]. Conversely, there are cases where functionally similar progenitors demonstrate differential immunophenotypes when derived from developmentally different sources [5] or whether the source is at steady-state or perturbed [12]. In the present study we developed a novel strategy for unbiased phenotypic identification of functionally distinct progenitors. By combining random, single-cell index sorting with clonal assays on custom lineage-permissive suspension culture system, we interrogate the functional identity of hundreds of progenitor cells. Each lineage potential is then linked back to the phenotype of the colony-initiating cell, deriving a unique composite immunophenotypic signature.

## Method development

**Fig. 1:**
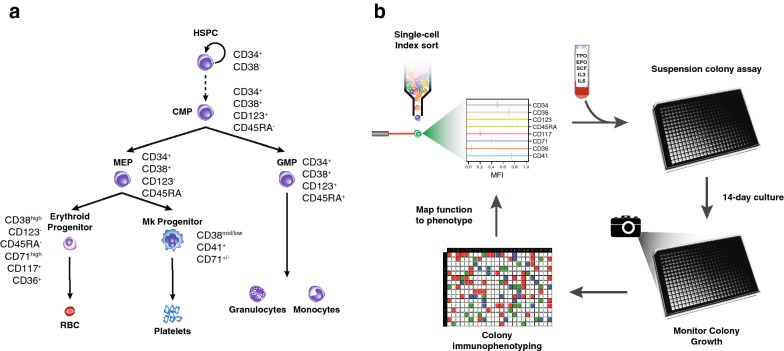
An unbiased approach for immunophenotypic identification of functionally distinct hematopoietic progenitors. **a** In the classical view of hematopoietic hierarchy progenitors are defined using complex nested gating strategies on defined immunophenotypic markers [2–4]. **b** In our approach we labelled a pool of CD34^+^ HSPCs with the currently established panel of markers and live, single events were index-sorted into an optically-clear 384-well. The progenitor potential of each cell was assessed in a custom colony assay suspension culture system, supplemented with cytokines that can support all myeloid lineages. Colonies were allowed to grow for 14 days and growth was monitored by an automated cell imager. Flow-cytometric analysis identified the type of colony which reflects the lineage potential of the sorted cell. The functional potential of the cell that initiated the colony was then mapped back to the composite phenotype of that cell

In order to validate the proposing strategy and test how it compares against the established purification protocols we applied the method to the discrimination of progenitors of the myeloid lineage (Fig. 1a). Hematopoietic stem and progenitor cells are found within the CD34^+^ fraction while further distinction between primitive and committed progenitors can be achieved by gating for different intensities along the CD38 axis. Most primitive multipotent progenitors (MPPs) can be found in the CD38^−^ fraction [13, 14], whereas more committed oligo- or unipotent progenitors are found within the CD38^+^ population [2]. Within the CD34^+^/CD38^+^, the addition of the CD123 (IL3Ra) and CD45RA can separate the more primitive common myeloid progenitor (CMP) (CD123^+^/CD45RA^−^) from its progeny, the oligopotent granulocytic–monocytic progenitor (GMP) (CD123^+^/CD45RA^+^) and the bipotent megakaryocytic–erythroid progenitor (MEP) (CD123^−^/CD45RA^−^) [2, 3]. Cells expressing the Kit ligand receptor c-Kit (CD117) have been functionally identified with a myelo-erythroid potential [15]. Addition of lineage-specific markers such as CD71, CD36, or CD41 can provide further enrichment for the committed erythroid and megakaryocytic progenitors, respectively [16, 17]. Starting with CD34^+^ enriched G-CSF mobilized peripheral blood mononuclear cells from healthy human donors, we labelled the cells with fluorochrome-conjugated antibodies against 8 markers (CD34, CD38, CD123, CD45RA, CD117, CD71, CD36, and CD41) and flow-sorted single, live events into an optically clear 384-well plate. The mean fluorescence intensity (MFI) for each of the 8 markers (Additional file 1: Figure S1) as well as the location of each individual cell in the 384-well plate is recorded and the cells are allowed to give rise to colonies for 14 days. In order to be able to detect all myeloid progenitors, we designed a suspension culture system based on serum-free media supplemented with a permissive cytokine cocktail containing SCF, IL-6, IL-3, SCF, EPO, and TPO (Additional file 2: Methods) which allows all myeloid lineages to emerge (Fig. 1b). Colony growth was monitored in frequent intervals using an automated cell imager (Additional file 3: Figure S2). Total clonal capacity of human adult CD34^+^ in the custom permissive media, calculated as the number of colonies above the selected cell number threshold on day 14, averaged to 28.6% (Additional file 3: Figure S2). After 14 days, detectable colonies were labelled for CD235a and CD41a, followed by flow-cytometry analysis. CD235a, the sialoglycoprotein glycophorin A, is the canonical marker of mature erythroid progenitors [18], while CD41 and CD42b are platelet glycoprotein expressed in primitive and mature megakaryocytes [19]. Distinction between purely erythroid or megakaryocytic, and mixed was primarily based on the ratio between CD41^+^ cells and CD235a^+^. Colonies where CD41^+^ frequency exceeds that of CD235a^+^ were labelled as megakaryocytic while those where CD235a^+^ cells were 0.2 × log2-fold the frequency of CD41^+^ were labelled as erythroid. Colonies where the ratio was anywhere between these values were identified as mixed. Finally, colonies negative for all markers were labelled as granulocytic/monocytic (GM) (Additional file 4: Figure S3a, b). Erythroid colonies were the most abundant accounting for 49.2% of all colonies observed with megakaryocytic following with 22.2%. Mixed colonies accounted for 15.9% of total, while granulocytic/monocytic were 12.7% (Additional file 4: Figure S3c). Upon colony identification, the MFI of each marker of the initiating cell was recalled and a continuous composite marker profile was assigned.

## Results and discussion

**Fig. 2:**
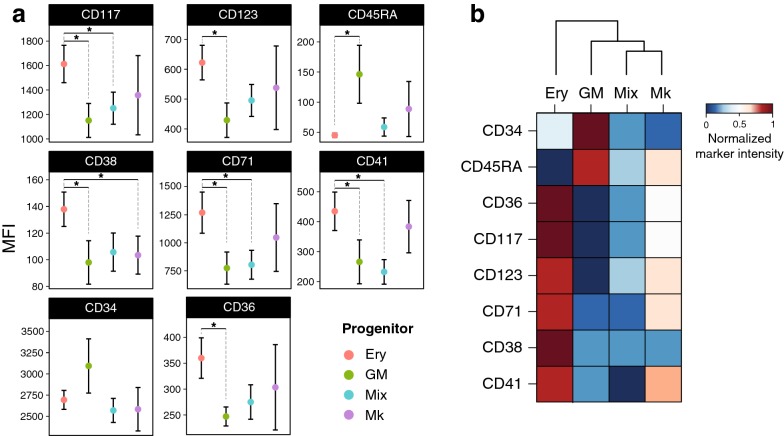
Functionally defined progenitors have unique composite immunophenotypes. **a** Four colony types can be detected in our colony-assay system, erythroid (Ery), granulocytic/monocytic (GM), megakaryocytic (Mk), and a mixture of all (Mix), each representing one functionally distinct progenitor. For each type of progenitor the mean and standard error of the MFI for each of the markers used is displayed, demonstrating immunophenotypic differences for each progenitor (*t test, *p* < 0.1). **b** Hierarchical clustering based on the average, center-scaled intensity of each marker can resolve the relationships of the four progenitors

The panel used in this experiment allowed us to confirm the identity of the progenitors and compare our results with previously reported data from the literature. For example, we confirm that progenitors with granulocytic/monocytic potential can be enriched by gating for CD45RA^+^ [2], as cells that gave rise to GM colonies in our assay exhibited significantly higher expression of CD45RA (Fig. 2a). All cells with erythroid restricted potential exhibited high levels of markers that distinguish erythroid progenitors such as CD117, CD71, and CD36. CD71 is one of the canonical markers used to identify erythroid cells, yet we found no statistically significant difference in CD71 expression between erythroid and megakaryocytic progenitors. Interestingly, Edvardsson et al. have also reported that cells with megakaryocytic potential can be found within the CD71^+^ population [20]. Likewise, CD41a marks cells with megakaryocytic capacity and indeed our experiments demonstrate that megakaryocytic progenitors exhibit high levels of CD41. However, cells with erythroid capacity also exhibited significantly high levels of CD41, in line with previous reports for shared features between the two progenitors [17]. Interestingly, we found erythroid progenitors to highly express CD123, an unexpected and contradictory result to the current knowledge. CD123 is a synonym for the Interleukin-3 receptor α-chain (IL-3Rα) which binds to the hematopoietic cytokine Interleukin-3 (IL-3) [21] inducing the proliferation of the myeloid lineages [22]. Historically, CD123 is used in the context CD34/CD38/CD45RA to enrich for either CMPs or GMPs [2] whereas little evidence is available on the expression of CD123 beyond myeloid and lymphoid lineages. Previous studies, report that CD123 negative cells within the CD34^+^ fraction from cord blood are enriched for erythroid progenitors, without however concluding on the erythroid capacity of CD123^+^ from G-CSF mobilized peripheral blood [23]. In order to validate our finding, we measured the expression of CD123 along the ex vivo human erythropoiesis. We confirmed that its expression increased after 6 days of differentiation a result in line with previous reports of the expression of CD123 during ex vivo erythropoiesis [24], thus identifying a novel marker able to distinguish erythroid progenitors (Additional file 5: Figure S4).

Conclusively, the above findings highlight the limitations associated with the established strategies for progenitor isolation. Most of the markers used here, with the exception of CD45RA do not follow a clear bimodal distribution to sufficiently distinguish between positive and negative cells (Additional file 1: Figure S1). Therefore, such discriminations are arbitrary and the definition can differ between laboratories or vary depending on donors or antibodies used, thus rendering the discrete gates rather problematic. Utilizing the above described 8-marker panel we were able to construct a composite, continuous, information-rich phenotypic signature able to resolve the relationships of the reported progenitors (Fig. 2b) according to the revised model of hematopoiesis [5]. We find that cells with Mk potential are closely related to the multipotent progenitors, concordant with the recent views that place the emergence of megakaryocytes at the multipotent progenitor compartment, while the erythrocytic and the granulocytic/monocytic lineages derive from unipotent progenitors. The proposed method can be applied to any progenitor cell with measurable functional output and can be further expanded using novel markers, or new combinations of established ones. The continuous marker intensities can also be translated to defined boolean gates in order to facilitate the flow-cytometric isolation of the progenitors with potentially higher functional purity. Furthermore, beyond the G-CSF mobilized peripheral blood derived CD34^+^ used here, additional hematopoietic sources can be evaluated (e.g. bone marrow, cord blood, fetal liver) as functional differences for immunophenotypically similar populations among sources have been previously demonstrated [25, 26]. This method can be particularly useful when robust identification methods are available like molecular screens. Such case can be the identification of leukemic progenitors where phenotypic and functional heterogeneity is common even among clones [27–29], but sequencing or PCR-based assays can provide unambiguous identification of leukemic cells which in combination with single-cell index sorting can reveal novel phenotypes for flow-cytometric estimation of the leukemic load. Beyond the phenotypic identification of leukemic cells, novel cell surface markers can also aid the development of monoclonal antibodies, targeting specifically the leukemic cells with the particular phenotype.

## Additional files

**Additional file 1: Figure S1.** Expression of cell surface markers from human adult CD34^+^ HSPCs. Cells are stained for CD34, CD38, CD45RA, CD123, CD117, CD71, CD36, and CD41. Then single, live events (light-blue shaded histograms) are sorted and the MFI for each of the markers for each cell sorted into the 384-well microplate is recorded. Grey shaded histograms is the total population acquired. Histograms are scaled to mode.

**Additional file 2.** Extended materials and methods along with detailed table with antibodies used, and data analysis details.

**Additional file 3: Figure S2.** Colony growth monitoring 14 days post single-cell index sorting. Colony growth is recorded for each well of the 384-well plate. Image on the left shows a well with > 500 cells. Cells are highlighted with green. Right, shows the cell count estimates from the automated cell imager and the threshold of 500 cells to distinguish wells with colonies versus wells with no detectable growth.

**Additional file 4: Figure S3.** Identification of colonies based on the expression of CD235a (*x* axis) and CD41 (*y* axis). **A**, **B** Distinction between erythroid, mixed and megakaryocytic colonies was made based on the ratio between CD41^+^ cells and CD235a^+^ cells. Colonies where CD41^+^ cells were more than CD235a were identified as megakaryocytic while colonies where CD235a^+^ cells where at least 1.15x more than CD41^+^ were identified as purely erythroid. Colonies where ratio between CD235a^+^ cells and CD41^+^ is proportional, were labelled as mixed. In addition, megakaryocytic colonies were distinguished from mixed on the frequency of CD42^+^ cells within the CD41^+^ fraction. Wells with > 80% double negative were identified as GM. **C** Frequency of each colony type as percentage of total colonies observed.

**Additional file 5: Figure S4.** Time-course of CD123 expression during the ex-vivo erythroid development from adult human CD34^+^. Expression of CD123 is increased from day 6 and on along with the emergence of erythroid progenitors in the culture. Top histogram, is Fluorescence Minus One (FMO) negative control.

## Data Availability

All data generated in this study are included in the main text and additional files.

## Abbreviations

HSPC: hematopoietic stem and progenitor cells
MPP: multi-potent progenitors
CMP: common myeloid progenitor
MEP: megakaryocytic-erythroid progenitor
GMP: granulocytic–monocytic progenitor
Ery: erythroid
Mk: megakaryocytic
GM: granulocytic–monocytic
MFI: mean fluorescence intensity
