# Supplementary figures and images for: Unbiased phenotypic identification of functionally distinct hematopoietic progenitors
Source: J Biol Res (Thessalon). 2019 Jul 18;26:4. doi: 10.1186/s40709-019-0097-7 (PMC6639971; doi:10.1186/s40709-019-0097-7)

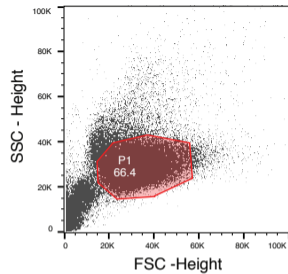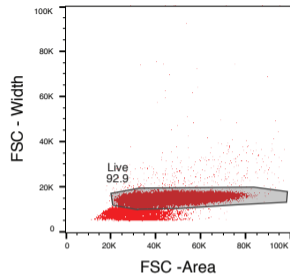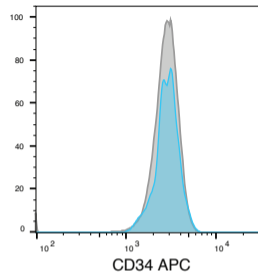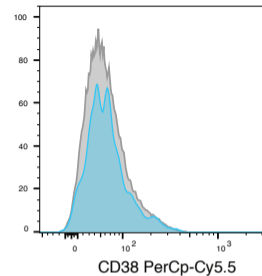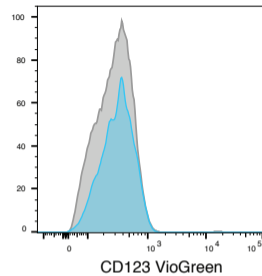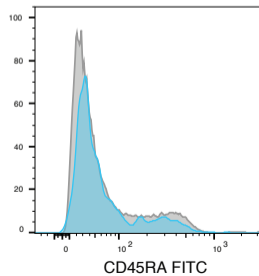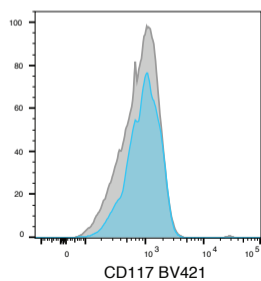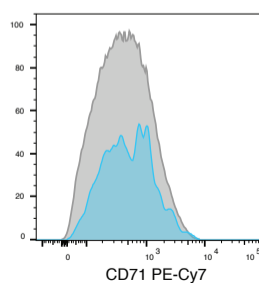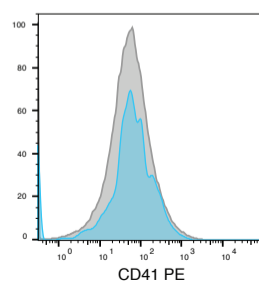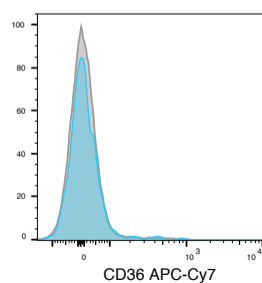

Supplement: Supplementary file 1 — Additional file 1: Figure S1. Expression of cell surface markers from human adult CD34+ HSPCs. Cells are stained for CD34, CD38, CD45RA, CD123, CD117, CD71, CD36, and CD41. Then single, live events (light-blue shaded histograms) are sorted and the MFI for each of the markers for each cell sorted into the 384-well microplate is recorded. Grey shaded histograms is the total population acquired. Histograms are scaled to mode. [file 40709_2019_97_MOESM1_ESM.pdf]

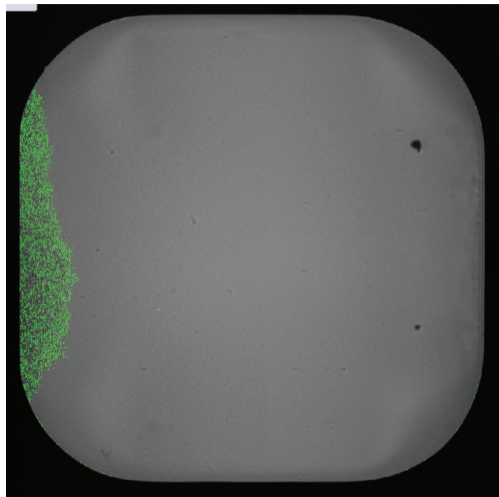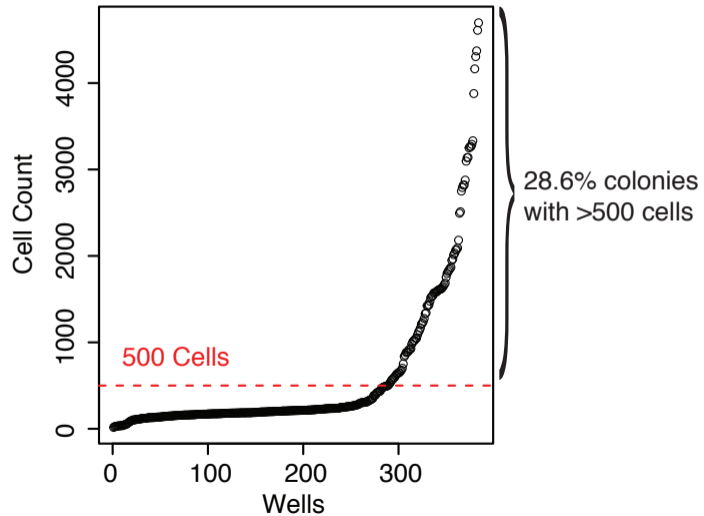

Supplement: Supplementary file 3 — Additional file 3: Figure S2. Colony growth monitoring 14 days post single-cell index sorting. Colony growth is recorded for each well of the 384-well plate. Image on the left shows a well with > 500 cells. Cells are highlighted with green. Right, shows the cell count estimates from the automated cell imager and the threshold of 500 cells to distinguish wells with colonies versus wells with no detectable growth. [file 40709_2019_97_MOESM3_ESM.pdf]

**A**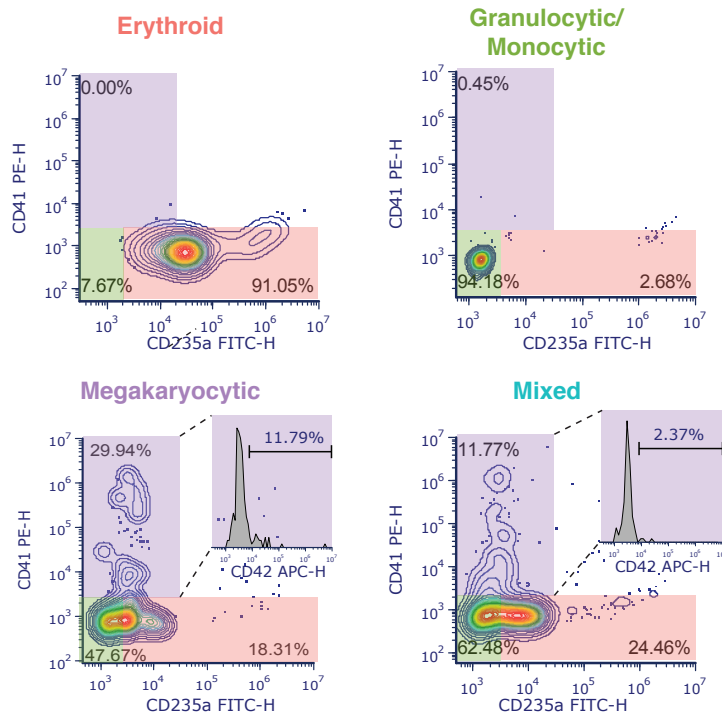**B**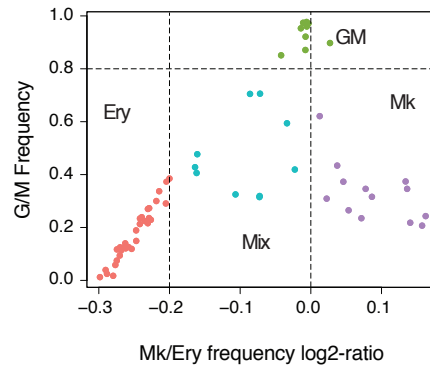**C**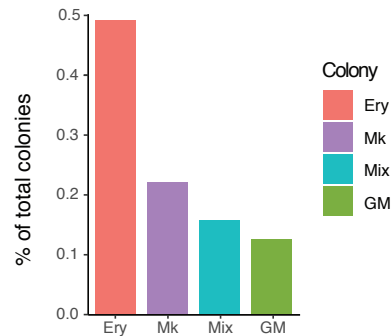

Supplement: Supplementary file 4 — Additional file 4: Figure S3. Identification of colonies based on the expression of CD235a (x axis) and CD41 (y axis). A, B Distinction between erythroid, mixed and megakaryocytic colonies was made based on the ratio between CD41+ cells and CD235a+ cells. Colonies where CD41+ cells were more than CD235a were identified as megakaryocytic while colonies where CD235a+ cells where at least 1.15x more than CD41+ were identified as purely erythroid. Colonies where ratio between CD235a+ cells and CD41+ is proportional, were labelled as mixed. In addition, megakaryocytic colonies were distinguished from mixed on the frequency of CD42+ cells within the CD41+ fraction. Wells with > 80% double negative were identified as GM. C Frequency of each colony type as percentage of total colonies observed. [file 40709_2019_97_MOESM4_ESM.pdf]

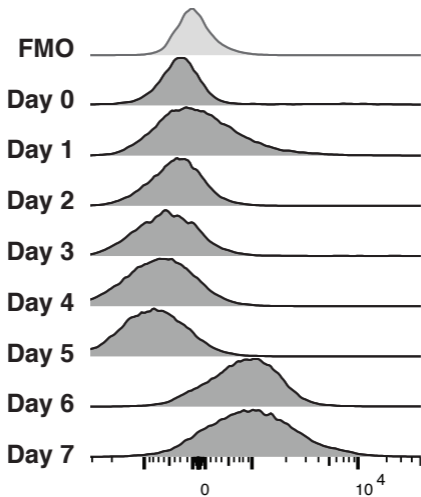

CD123 APC-A700-A

Supplement: Supplementary file 5 — Additional file 5: Figure S4. Time-course of CD123 expression during the ex-vivo erythroid development from adult human CD34+. Expression of CD123 is increased from day 6 and on along with the emergence of erythroid progenitors in the culture. Top histogram, is Fluorescence Minus One (FMO) negative control. [file 40709_2019_97_MOESM5_ESM.pdf]
