# Supplementary material for: Unbiased phenotypic identification of functionally distinct hematopoietic progenitors
Source: J Biol Res (Thessalon). 2019 Jul 18;26:4. doi: 10.1186/s40709-019-0097-7 (PMC6639971; doi:10.1186/s40709-019-0097-7)
Supplement: Supplementary file 2 — Additional file 2. Extended materials and methods along with detailed table with antibodies used, and data analysis details. [file 40709_2019_97_MOESM2_ESM.docx]

**Extended Materials and Methods**

**Cell Culture**

Cryopreserved G-CSF-mobilized, CD34+ enriched peripheral blood mononuclear cells from healthy adult human donors purchased from Fred Hutch CCEH - Hematopoietic Cell Procurement and Processing Services (Seattle, WA, USA) were thawed rapidly at 37 ºC, resuspended in PBS + 0.5% BSA and centrifuged at 250 *x* g for 10 min. The cell pellet is then resuspended in serum-free media containing StemSpan H3000 (StemCell Technologies, Vancouver BC, Canada), supplemented with 1x penicillin/streptomycin (Gibco) and 1x StemSpan CC110 (StemCell Technologies, Vancouver BC, Canada) and allowed to recover overnight before transfer to primary culture or index sorting. For the custom permissive media, single cells were sorted into an optically clear 384-well microplate (Greiner-Bio) containing 60 µL of serum-free base media StemSpan H3000 (StemCell Technologies, Vancouver BC, Canada) supplemented with 25 ng/mL Stem Cell Factor (SCF), 10 ng/mL Interleukin-3 (IL-3), 10 ng/mL Interleukin-6 (IL-6), 3 IU/mL Erythropoietin (EPO), and 50 ng/mL Thrombopoietin (TPO). During the 14-day culture, media were added to wells with evident evaporation to avoid “edge-effects”. Colony growth was monitored using the Celigo Imaging Cytometer (Nexcelcom Bioscience).

Human erythroid differentiation was induced *ex vivo* from recovered CD34+ cells for 12 days according to the protocol developed the Douay L. group [1]. Briefly, cells for the first 7 days were cultured in serum-free IMDM-based media in the presence of 3 IU/mL EPO, 5 ng/mL IL-3, 100 ng/mL SCF, and 1 µM Hydrocortisone, supplemented with 5% human plasma, 2 U/mL heparin, 10 µg/mL insulin (Sigma), and 330 µg/mL human Holo-Transferrin (Sigma). After 7 days, IL-3 and Hydrocortisone are withdrawn from the media and cell culture is continued for 5 more days. All cytokines were purchased from PeproTech (Rocky Hill, NJ, USA).

**Flow Cytometry**

For index sorting, cells are harvested and washed with PBS + 0.5% BSA. The cell pellet is then resuspended in staining buffer (PBS + 0.5% BSA) containing CD34 APC, CD38 PerCP-Cy5.5, CD45RA FITC, CD123 VioGreen, CD117 BV421, CD71 PE-Cy7, CD36, APC-Cy7, and CD41 PE (ST1) in final concentration as per manufacturer’s instructions. Cells are incubated at 4 ºC for 30min and then washed and resuspended in staining buffer. The sample is then acquired with MoFlo Astrios EQ (Beckman Coulter) and single-cells are sorted into the 384-well microplate. The MFI for each marker and the well for each cell are exported in a table. Bulk population analysis was performed using FlowJo^TM^10 (Beckton Dickinson).

For colony phenotyping, CD235a FITC, CD41 PE, and CD42b APC (ST1) were added directly to the microplate well in a volume proportional to the well volume so that final antibody concentration is according to manufacturer’s recommendations, without prior washing of the cells. After 30 minutes incubation at room temperature, staining buffer was added to the well for final volume of 250 µL. Samples were then acquired using CytoFLEX S (Beckman Coulter) and analyzed using a demo version of FCS Express 6 (De Novo Software).

To test IL3Ra expression during *ex vivo* erythropoiesis, primary erythroid culture was sample daily for the first 6 days and cells were washed with staining buffer. Pellet was resuspended in 50 µL staining buffer containing 5 µL of CD123 Alexa Fluor 700 (ST1) and incubated for 30 min at 4 ºC. Cells were then washed with staining buffer and the pellet was resuspended in staining buffer containing 1% paraformaldehyde and stored at 4 ºC until flow cytometric analysis. Samples were acquired using CytoFLEX S (Beckman Coulter). Analysis was performed using FlowJo^TM^10 (Beckton Dickinson).

**Table 1.** List of antibodies used.

| **Antibody** | **Conjugate** | **Host/**  **Specificity** | **Isotype** | **Clone** | **Company** | **Catalog Number** |
| --- | --- | --- | --- | --- | --- | --- |
| CD34 | APC | Mouse α-Human | IgG1 κ | 581 | BD Biosciences | 560940 |
| CD38 | PerCP-Cy5.5 | Mouse α-Human | IgG1 κ | HIT2 | BD Biosciences | 561106 |
| CD117 | Brilliant Violet 421 | Mouse α-Human | IgG1 κ | 104D2 | BioLegend | 313215 |
| CD45RA | FITC | Mouse α-Human | IgG2b κ | HI100 | BioLegend | 304106 |
| CD41 | PE | Mouse α-Human | IgG1 κ | HIP8 | BD Biosciences | 555467 |
| CD42 | APC | Mouse α-Human | IgG1 κ | HIP1 | BD Biosciences | 551061 |
| CD71 | PE-Cy7 | Mouse α-Human | IgG2a κ | CY1G4 | BioLegend | 334111 |
| CD123 | VioGreen | Mouse α-Human | IgG2a κ | AC145 | Miltenyi Biotec | 130‑106‑711 |
| CD123 | Alexa Fluor 700 | Mouse α-Human | IgG1 κ | 6H6 | BioLegend | 306039 |
| CD36 | APC-Cy7 | Mouse α-Human | IgG2a κ | 5-271 | BioLegend | 336213 |
| CD235a | FITC | Mouse α-Human | IgG2b κ | GA-R2 (HIR2) | BD Biosciences | 559943 |

**Data Analysis**

Statistical analysis was performed using R [2]. Differences in expression for each marker between progenitors was performed using a two-way Student’s t-test on the MFI values and significance was inferred from the Bonferroni corrected p-values. Significance threshold was set as p < 0.1, throughout. Hierarchical clustering analysis between progenitors was performed on Euclidean distance of the center-scaled MFI values and a complete linkage tree was generated.

**References**

1. Giarratana M-C, Kobari L, Lapillonne H, Chalmers D, Kiger L, Cynober T, et al. Ex vivo generation of fully mature human red blood cells from hematopoietic stem cells. Nat Biotechnol. 2005; doi:10.1038/nbt1047.

2. R Core Team. R: A Language and Environment for Statistical Computing. Vienna, Austria: R Foundation for Statistical Computing; 2013.
